# *Drosophila* TET acts with PRC1 to activate gene expression independently of its catalytic activity

**DOI:** 10.1126/sciadv.adn5861

**Published:** 2024-05-03

**Authors:** Guerric Gilbert, Yoan Renaud, Camille Teste, Nadège Anglaret, Romane Bertrand, Sven Hoehn, Tomasz P. Jurkowski, Bernd Schuettengruber, Giacomo Cavalli, Lucas Waltzer, Laurence Vandel

**Affiliations:** ^1^ Université Clermont Auvergne, CNRS, INSERM, iGReD, F-63000 Clermont-Ferrand, France.; ^2^ Cardiff University, School of Biosciences, Museum Avenue, CF10 3AX Cardiff, Wales, UK.; ^3^ Institute of Human Genetics, UMR9002, CNRS and University of Montpellier, Montpellier, France.

## Abstract

Enzymes of the ten-eleven translocation (TET) family play a key role in the regulation of gene expression by oxidizing 5-methylcytosine (5mC), a prominent epigenetic mark in many species. Yet, TET proteins also have less characterized noncanonical modes of action, notably in *Drosophila*, whose genome is devoid of 5mC. Here, we show that *Drosophila* TET activates the expression of genes required for larval central nervous system (CNS) development mainly in a catalytic-independent manner. Genome-wide profiling shows that TET is recruited to enhancer and promoter regions bound by Polycomb group complex (PcG) proteins. We found that TET interacts and colocalizes on chromatin preferentially with Polycomb repressor complex 1 (PRC1) rather than PRC2. Furthermore, PRC1 but not PRC2 is required for the activation of TET target genes. Last, our results suggest that TET and PRC1 binding to activated genes is interdependent. These data highlight the importance of TET noncatalytic function and the role of PRC1 for gene activation in the *Drosophila* larval CNS.

## Teaser

The epigenetic enzyme TET recruits PRC1 to activate transcription in a catalytic-independent manner.

## INTRODUCTION

Epigenetic enzymes are involved in reversible modifications of DNA or histones, which affect chromatin structure and recruitment of chromatin binding factors (*1*). Thereby, they play an important role in genome biology notably by regulating gene transcription, DNA replication, or genome stability. Accordingly, the balanced action of writers, which deposit epigenetic modifications and erasers, which remove these marks, controls cell fate determination and differentiation in multicellular organisms. Mutations affecting these enzymes can cause developmental defects and various pathologies, including cancers and neurodegenerative disorders (*2*, *3*). Drugs targeting the enzymatic activity of these factors have thus emerged as promising therapeutics (*4*). Yet, recent studies revealed that several epigenetic enzymes also have crucial catalytic-independent functions (*5*), calling for a careful reexamination of the bases for their loss-of-function phenotypes.

Since 2009, members of the ten-eleven translocation (TET) family have emerged as key players in the epigenetic regulation of gene expression (*6*). TET proteins are Fe^2+^- and 2-oxoglutarate–dependent dioxygenases capable of oxidizing 5-methylcytosine (5mC) on DNA into 5-hydroxymethylcytosine (5hmC) and further oxidized derivatives, which serve as intermediate products in the cytosine demethylation cascade or as stable epigenetic marks with distinct regulatory roles (*7*–*11*). As 5mC is a prevalent epigenetic mark in many species (*12*), the discovery of 5mC “demethylases” has sparked much interest. TET enzymes are conserved across evolution in metazoans, and their functions have been particularly well studied in mammals, where the three TET paralogs (TET1 to TET3) are implicated in a variety of biological processes including regulation of embryonic stem cell pluripotency, blood cell differentiation, and immune response or nervous system development and neuronal plasticity (*13*). Their role is also highlighted in human pathologies as somatic mutations affecting *TET* genes are frequently associated with the development of various cancers, in particular leukemia (*14*), and germline mutations have been linked to blood cell transformation (*15*, *16*) or neurodevelopmental and neurodegenerative disorders (*17*–*19*).

So far, TET protein activity has been largely associated with their capacity to oxidize 5mC, thereby regulating transcription by promoting DNA hypomethylation (*6*). Consistently, most studies focused on the link between TET and 5mC/5hmC levels, in particular in pathological contexts such as leukemia or neurodevelopmental and neurodegenerative disorders (*13*, *14*). Still, less-studied noncanonical modes of actions have been described that contribute to TET functions (*6*). First, TET can oxidize methylcytosines on RNA (m5C) (*20*), and it was proposed that TET-mediated hydroxymethylation of mRNA (*6*, *21*, *22*) or tRNA (*23*, *24*) regulates gene expression at the posttranscriptional level. Second, it was shown in vertebrates that some TET functions are independent of their enzymatic activity (*6*, *25*–*27*). TET proteins also control gene expression by interacting with other factors implicated in chromatin regulation (*6*). For instance, recent evidence showed that TET1 can repress gene expression in a catalytic-independent manner by recruiting the Polycomb repressive complex 2 (PRC2) and the Sin3A deacetylase to target histone H3 lysine 27 (H3K27) modifications in embryonic stem cells (*26*, *28*).

The *Drosophila* genome contains a single and well-conserved *Tet* gene although it does not contain 5mC DNA methyltransferase genes and is largely devoid of this epigenetic mark (*29*, *30*). This insect thus stands as a valuable model system to investigate TET noncanonical functions. The complete loss of *Tet* expression is lethal at the pupal stage (*31*–*33*), indicating that TET plays a vital role in this organism. Further analyses showed that *Tet* loss or knockdown affects ovarian development (*32*, *33*), zygotic genome activation in the early embryo (*34*), larval locomotion (*32*), as well as larval and adult brain development (*31*, *35*–*37*). At the molecular level, TET function was attributed either to the oxidation of m5C on mRNA to promote translation (*31*) or to the oxidation of 6-methyladenine (6mA) on DNA to control transcription (*33*, *37*). However, the direct impact of TET enzymes on mRNA modifications remains disputed notably because hm5C is essentially detected on tRNAs (*23*, *24*, *37*). In addition, the presence and potential significance of 6mA in metazoan genomes remain highly controversial (*38*), and the capacity of *Drosophila* TET to oxidize 6mA is at odds with the conserved structure of its catalytic domain (CD), which lacks crucial amino acids necessary for 6mA recognition and oxidation in more distant TET homologs or the related alkane hydroxylase AlkB family of 6mA demethylase (*39*, *40*). Along that line, our recent results support the conclusion that TET does not act as a 6mA demethylase in *Drosophila* and showed that, in contrast to TET expression, its catalytic activity is dispensable for adult fly emergence, survival, and reproduction (*41*). Thus, TET essentially acts in a catalytic-independent manner in *Drosophila*, but the underlying molecular mechanisms remain unknown.

To fill this gap, we investigated the TET mode of action in the larval central nervous system (CNS). By comparing the phenotypes associated with the absence of TET expression or only of its catalytic activity and using a combination of genetics, transcriptomics, and chromatin profiling, we show that TET can directly activate gene transcription independently of its catalytic activity. Our results show that TET collaborates with Polycomb components for its gene regulatory activity. Polycomb group (PcG) proteins are organized into two main complexes, the Polycomb repressor complex 1 (PRC1) and PRC2, which are well known for their function in the maintenance of a repressive chromatin state, with PRC2 depositing the H3K27me3 repressive mark and PRC1 the H2AK118ub mark (*42*, *43*). PcG complexes are also found in active chromatin regions, and several lines of evidence indicate that PRC1 can act independently of PRC2 to activate gene expression, in particular in *Drosophila* (*44*–*49*). Our data show that TET preferentially colocalizes with PRC1 rather than with PRC2 on chromatin and that TET interacts with PRC1 but not with PRC2 in the larval CNS. Moreover, it appears that PRC1 is required for the activation of TET target genes, while PRC2 is dispensable. In addition, we found that TET and PRC1 facilitate each other’s recruitment to chromatin on TET-activated target genes. Our results highlight a hitherto unknown mode of gene expression regulation by TET, which does not require its catalytic activity, and support a role for PRC1 in the activation of gene transcription independently of PRC2.

## RESULTS

### TET controls larval CNS development mainly in a catalytic-independent manner

**Fig. 1.**
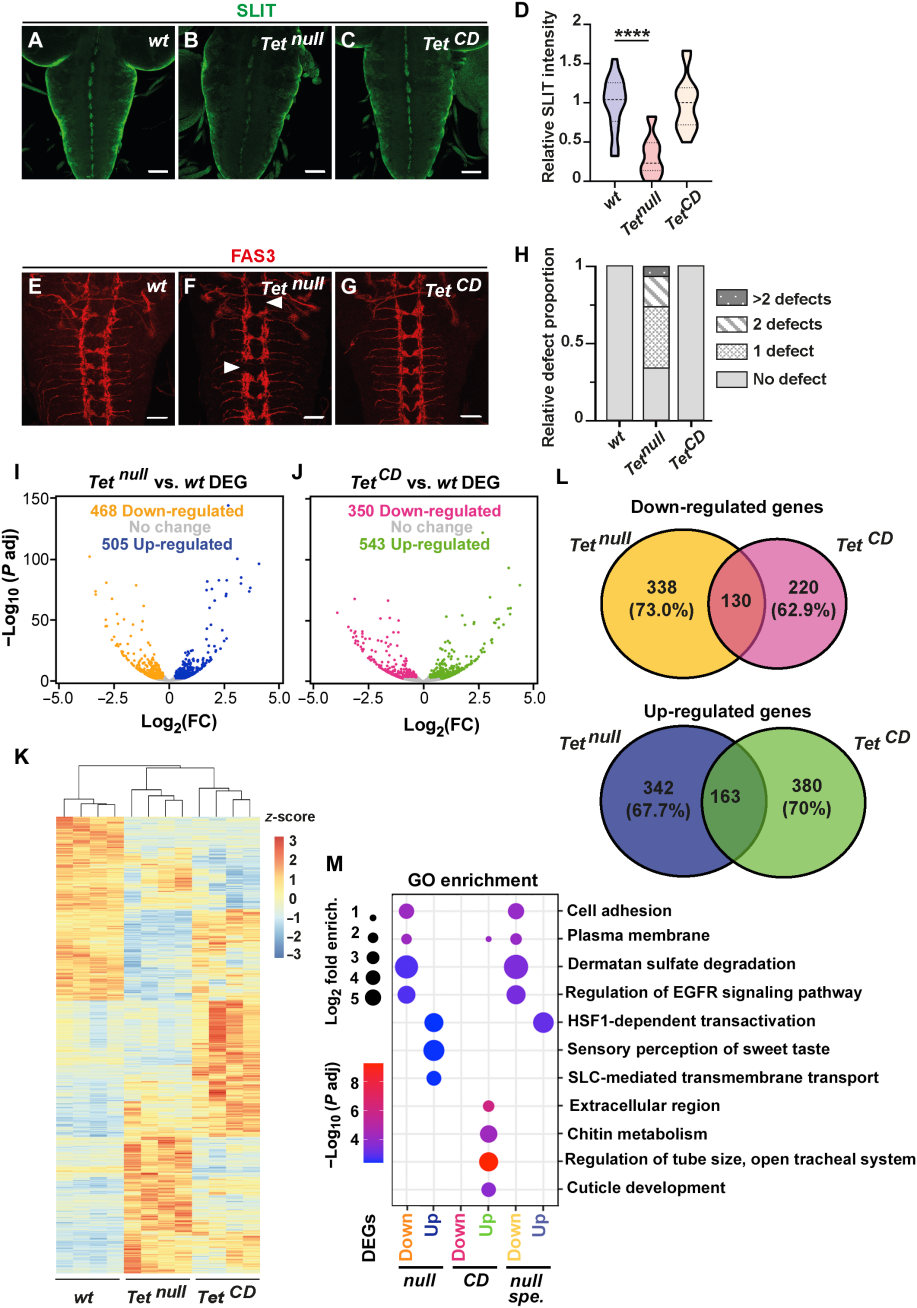
TET controls larval CNS development mainly in a catalytic-independent manner. (**A** to **C**) Immunostaining showing Slit expression in the VNC of wild-type (A, *wt*), *Tet^null^* (B), and *Tet^CD^* (C) third instar larvae. Scale bars, 50 μm. (**D**) Quantifications of Slit expression levels (*n* = 15 larvae per genotype). One-way analysis of variance (ANOVA), ****P < 0.0001. (**E** to **G**) Immunostaining of Fas3 expression in the VNC of wild-type (E), *Tet^null^* (F), and *Tet^CD^* (G) third instar larvae. Interruptions in the axonal chain are indicated by white arrowheads. Scale bars, 50 μm. (**H**) Quantifications of the proportion of larvae with axonal chain interruptions (*n* = 20 larvae per genotype). (**I** and **J**) Volcano plots illustrating differentially expressed genes [DEG; adjusted *P* value < 0.01; fold change (FC) > 1.2] between wild-type and *Tet^null^* (I) or *Tet^CD^* (J) CNS as determined by RNA-seq. (**K**) Heatmap and hierarchical clustering of the differentially expressed genes for the indicated genotypes. (**L**) Venn diagrams representing the overlap between the genes either down-regulated (top) or up-regulated (bottom) in the absence of TET expression (*Tet^null^*) or of its catalytic activity (*Tet^CD^*). (**M**) Main Gene Ontology (GO) categories overrepresented among all the genes down- or up-regulated in either *Tet^null^* or *Tet^CD^* and those deregulated specifically in *Tet^null^* but not in *Tet^CD^* (*null spe*.). HSF1, Heat Shock Transcription Factor 1; SLC, Solute-Carrier.

To analyze TET catalytic and noncatalytic functions in the larval CNS, we made use of two alleles: *Tet^null^* (*31*), which carries a clean deletion abolishing the transcription of all *Tet* isoforms, and *Tet^CD^* (for *Tet Catalytic Dead*) (*41*), which carries a double point mutation (HrD to YrA) in the conserved iron binding motif required for the catalytic activity of TET/AlkB family of enzymes (*50*). Consistently, in vitro enzymatic assays showed that the recombinant CD of *Drosophila* TET carrying this point mutation was unable to oxidize 5mC into 5hmC, whereas we observed efficient oxidation using the wild-type counterpart (fig. S1). Besides, unlike *Tet^null^* homozygous, which die at the pupal stage, *Tet^CD^* flies are viable (*41*), indicating that TET enzymatic activity is largely dispensable for fly development. It was shown that RNA interference (RNAi)–mediated *Tet* knockdown or allelic combinations causing a strong decrease in *Tet* expression led to a reduction in the expression of the axon guidance ligand Slit (*35*, *36*), which is normally expressed in midline glial cells of the larval CNS. Consistent with the role of Slit, this reduction was associated with axon guidance defects in the ventral nerve cord (VNC) as revealed by immunostaining against the adhesion molecule Fasciclin 3 (Fas3) (*35*, *36*). In line with these observations, Slit levels were strongly decreased in the VNC of *Tet^null^* larvae as compared to control (Fig. 1, A, B, and D). However, they were not affected in *Tet^CD^* (Fig. 1, A, C, and D). Similarly, Fas3 immunostainings revealed that longitudinal axon projections were interrupted (white arrowheads) in ~66% of the cases in the absence of *Tet* expression, while they were not affected in *Tet^CD^* larvae or wild-type conditions (Fig. 1, E to H). These results indicate that TET controls Slit expression and axonal projections independently of its enzymatic activity.

To gain deeper molecular insights into the possible catalytic and noncatalytic functions of TET, we established the genome-wide expression profile of *Tet^null^*, *Tet^CD^*, and wild-type third instar larval CNS by RNA sequencing (RNA-seq). Differential gene expression analyses showed that complete TET loss was associated with the activation of the expression of 505 genes and the down-regulation of 468 genes (Fig. 1I and table S1). Similarly, the loss of TET catalytic activity led to the up-regulation of 543 genes and the down-regulation of 350 genes (Fig. 1J and table S2). Hierarchical clustering and Venn diagram analyses showed a distinct pattern of gene deregulation between these two mutant conditions, with limited overlap between the genes either up- or down-regulated in the CNS of *Tet^null^* and *Tet^CD^* larvae (Fig. 1, K and L). Hence, loss of TET expression or of its catalytic activity leads to distinct transcriptome changes. Around 70% (680 of 973) of the genes deregulated in *Tet^null^* are not affected in *Tet^CD^* (Fig. 1L), indicating that TET controls gene expression of many genes in a catalytic-independent manner in the larval CNS.

Consistently, gene set enrichment identified distinct signatures between gene sets deregulated in *Tet^null^* or *Tet^CD^* (Fig. 1M and table S3). For instance, up-regulated genes were associated with sensory perception of sweet taste, heat response, or transmembrane transport in *Tet^null^*, whereas they were implicated in tracheal and cuticle development or chitin and cuticle metabolism in *Tet^CD^*. Down-regulated genes were associated with cell adhesion, dermatan sulfate degradation, or epidermal growth factor receptor (EGFR) signaling pathway in *Tet^null^*, while no notable enrichment was found in *Tet^CD^*. The enriched terms in *Tet^null^* were essentially contributed by genes deregulated in a catalytic-independent manner (i.e., genes affected in *Tet^null^* only) (Fig. 1M and table S3). For instance, of the 10 genes of the EGFR pathway down-regulated in *Tet^null^*, 9 were not affected in *Tet^CD^* and 8 coded for negative regulators, suggesting that TET contributes in an enzymatic-independent manner to the regulation of the EGFR pathway, which is important for ventral midline glial cells development (*51*, *52*). Reminiscent of the projection defects observed specifically in *Tet^null^* larvae (Fig. 1, E to H), many genes implicated in axonal guidance including *slit* as well as *Ephrin* and *Ephrin receptor tyrosine kinase (Eph)* (*53*), *failed axon connections* (*fax*) (*54*), *Fasciclin 2* (*Fas2*) (*55*), *Laminin A* (*LanA*) (*56*), or *off-track* (*otk*) (*57*) were down-regulated in *Tet^null^* but not in *Tet^CD^* (tables S1 and S2).

In sum, these results show that the loss of TET enzymatic activity does not recapitulate TET total loss of function in the larval CNS as *Tet^CD^* and *Tet^null^* mutations elicit distinct changes. Our data also suggest that TET expression rather than its catalytic activity is key for the proper activation of genes implicated in CNS development.

### TET binds to enhancers and active transcriptional start sites to activate gene expression in a catalytic-independent manner

**Fig. 2.**
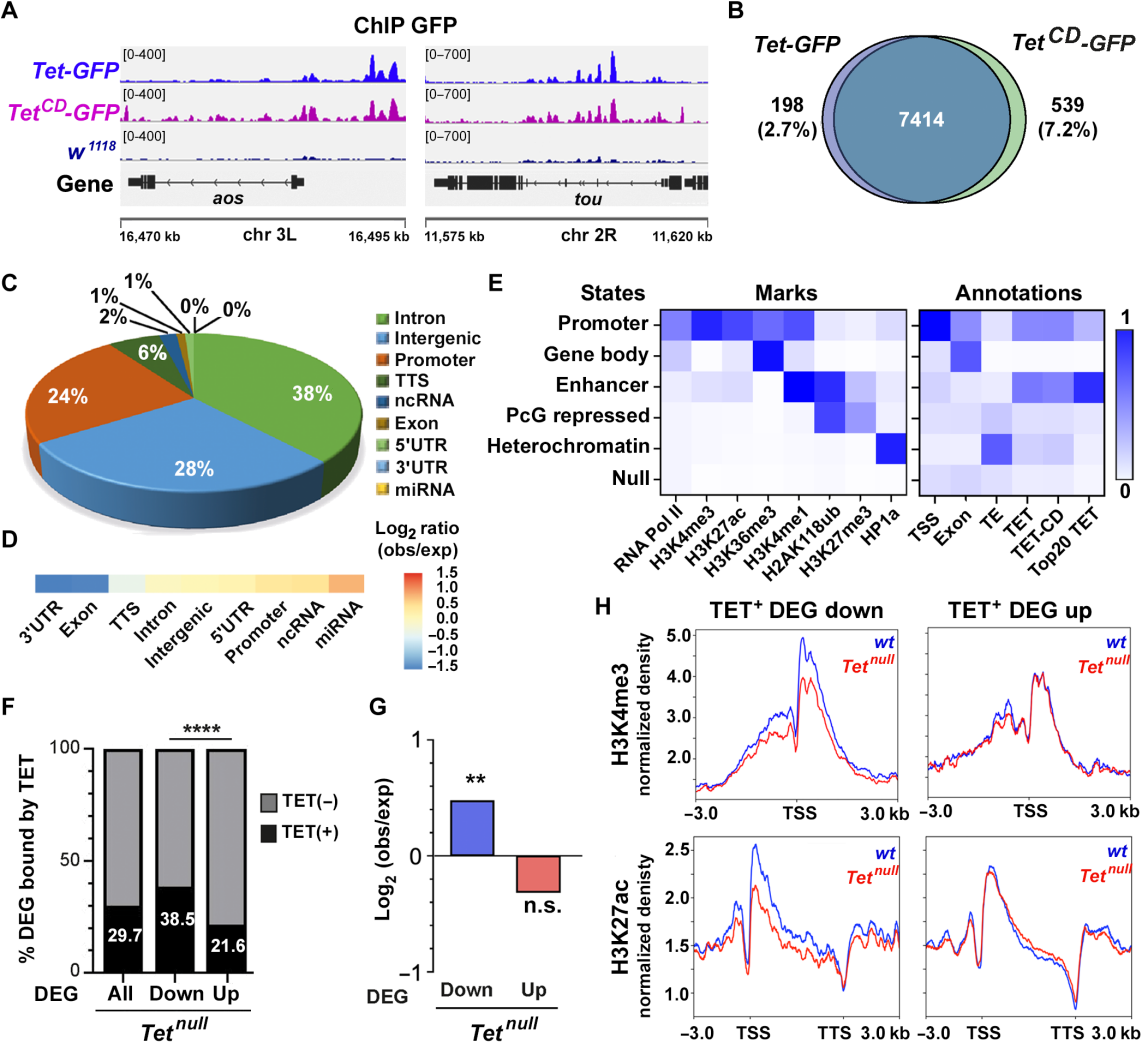
Genome-wide binding profile of TET to chromatin in the larval CNS. (**A**) Integrative genomics viewer (IGV) tracks showing peaks of anti-GFP ChIP-seq on dissected CNS from *TET-GFP*, *TET^CD^-GFP*, or *w^1118^* third instar larvae. (**B**) Venn diagrams of the overlap between TET-GFP and TET^CD^-GFP peaks. (**C**) Pie chart of TET-GFP peaks distribution according to RefSeq genomic annotations. (**D**) Heatmap showing TET-GFP enrichment according to genomic features. (**E**) Enrichment of TET-GFP, TET^CD^-GFP, and the top 20% TET-GFP peaks to the different chromatin states or genomic annotations of the larval CNS as defined by ChromHMM using ChIP-seq or CUT&RUN signals for the indicated factors/histone marks (as described in Materials and Methods). TE, transposable elements. (**F**) Proportions of differentially expressed genes (DEG) between wild-type and *Tet^null^* larval CNS with TET peaks within 2 kb of their TSS or in their gene body (TET^+^). (**G**) Enrichment of TET peaks among up- or down-regulated genes as compared to random sets of genes. (F and G) Fisher’s exact test, **P < 0.01; ****P < 0.0001. (**H**) Profiles of H3K4me3 (top) or H3K27ac (bottom) CUT&RUN signals on differentially expressed TET^+^ target genes in the CNS of *wt* (blue) or *Tet^null^* (red) larvae. 3′UTR, 3′ untranslated region; miRNA, microRNA; n.s., not significant.

Next, to characterize the TET mode of action, we sought to determine its genomic binding profile. Accordingly, we performed chromatin immunoprecipitations followed by high-throughput sequencing (ChIP-seq) on dissected third instar larval CNS from *Tet-GFP* or *Tet^CD^-GFP* knock-in flies as well as *w^1118^* flies (as negative control) using an anti-GFP antibody (Fig. 2A). We detected 7612 binding sites for wild-type TET-GFP fusion protein and 7953 for its catalytic dead counterpart, with more than 92% of overlap between the two sets (Fig. 2B), indicating that the HrD to YrA mutation has a minor impact on TET binding site repertoire. As compared to NCBI Reference Sequence database (RefSeq) *Drosophila melanogaster* genomic regions, around 38% of TET peaks localized in introns, 28% in intergenic regions, and 24% in promoters (Fig. 2C). No notable difference was observed with TET CD (fig. S2A). Moreover, we observed a slight overrepresentation for TET peaks at promoter regions as well as at noncoding RNA (ncRNA) and microRNA genes (Fig. 2D). Conversely, TET was underrepresented in 3′ untranslated regions, exons, and transcription termination sites (TTS). Of note, similar over- and underrepresentations were found for TET CD peaks (fig. S2B).

To analyze the signature of TET binding sites within the context of the larval CNS chromatin, we performed ChIP-seq or cleavage under targets and release using nuclease (CUT&RUN) for the following histone marks: H3K4me3, H3K27ac, H3K27me3, and H2AK118ub. In addition, we collected public datasets for H3K4me1 (*47*), H3K36me3 (*58*), RNA polymerase II (*58*), and the heterochromatin-associated protein HP1a (*59*) (see Materials and Methods). We then performed a chromatin state discovery and genome annotation analysis using the multivariate hidden Markov model trained on histone modifications to identify chromatin states (ChromHMM) (*60*). With a six-state model, we found that TET essentially targets enhancers (42% of TET peaks) and active transcription start site (TSS)/promoter regions (17% of TET peaks) (Fig. 2E and fig. S3). Again, a similar distribution was observed with TET CD. In addition, focusing on the top 20% TET peaks, we also observed that TET primarily binds enhancers (Fig. 2E and fig. S3).

The integration of ChIP-seq and RNA-seq results showed that 29.7% (289 of 973) of the genes deregulated in *Tet^null^* have a TET binding site within 2 kb of their TSS or in their gene body (Fig. 2F), with TET peaks significantly more frequently found in down-regulated than in up-regulated genes (38.5% versus 21.6%, *P* value < 0.001) (Fig. 2F). Also, as compared to random gene sets, TET peaks were overrepresented in down-regulated genes but not in up-regulated ones (Fig. 2G), suggesting that TET may play a more prominent role in gene activation than gene repression. In agreement, upon loss of TET, we observed a reduction in the levels of active histone marks, H3K4me3 and H3K27ac, at down-regulated TET-bound genes, suggesting that TET contributes to their transcriptional activation. In contrast, H3K4me3 and H3K27ac profiles of up-regulated TET targets were not affected in *Tet^null^* larvae, suggesting that their regulation is not transcriptional (Fig. 2H). Similar experiments in *Tet^CD^* larvae showed that the loss of TET enzymatic activity did not alter the H3K4me3 profile of either up or down-regulated TET targets (fig. S4). Together, these data suggest that TET can directly contribute to gene transactivation in a catalytic-independent manner.

### TET preferentially colocalizes with PRC1-bound regulatory regions

**Fig. 3.**
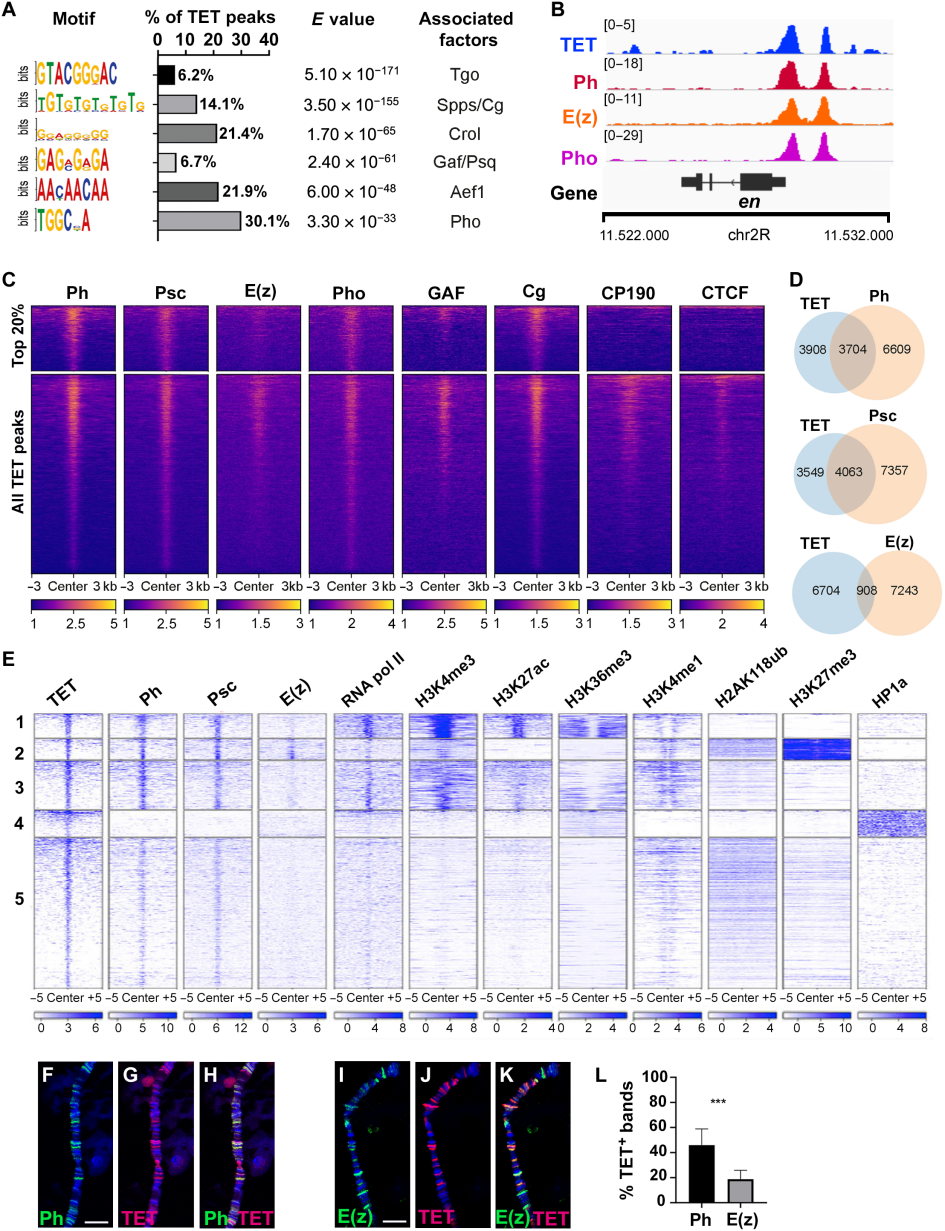
TET colocalizes with PRC1 and PRC2. (**A**) De novo motif discovery analysis at TET binding sites with their abundance and the associated factors. (**B**) IGV ChIP-seq tracks showing the binding of TET, Ph, E(z), and Pho on the *engrailed* (*en*) locus in the CNS of third instar larvae. (**C**) Heatmaps of ChIP-seq signals for Polycomb subunits [Ph, Psc, and E(z)], Polycomb recruiters (Pho, GAF, and Cg), or insulators (CP190 and CTCF) on all TET peaks (±3 kb of the center) or the top 20% of TET peaks in third instar larval CNS. (**D**) Venn diagrams of the overlaps of TET peaks with Ph, Psc, or E(z) peaks assessed between MACS2-defined narrow peaks. (**E**) Heatmap showing ChIP-seq or CUT&RUN signals for various proteins or histone modifications surrounding TET binding sites ±5 kb in third instar larval CNS. K-mean clustering was performed using histone marks as well as RNA pol II and HP1a signals. (**F** and **I**) Immunostainings on salivary gland polytene chromosomes of *sgs3-GAL4; UAS-HA-TET* third instar larvae showing the expression of Ph (F) or E(z) (I) and a hemagglutinin (HA)–tagged version of TET (**G** and **J**). (**H** and **K**) Merged panels. DNA was stained with DAPI (blue). Scale bars, 10 μm. (**L**) Quantifications of the colocalization between HA-TET–positive bands and Ph or E(z) immunostaining on 10 polytene chromosome regions. Student’s *t* test, ***P < 0.001.

Next, we performed de novo motif discovery analyses at TET peaks. Besides a very significant enrichment for Tango consensus binding sites (present in 6% of TET peaks, overrepresentation *E* value = 5.1 × 10^−171^), we primarily recovered motifs corresponding to previously assigned binding sites for the transcription factors Pleiohomeotic (Pho) (30%), Crooked-legs (21%), Combgap (Cg), and Specificity Protein 1 (Sp1) for pairing-sensitive silencing (Spps) (14%) or GAGA factor (GAF) and Pipsqueak (Psq) (7%), which are well-established recruiters for PcG proteins (Fig. 3A) (*43*, *44*, *61*). In addition, 22% of TET peaks were associated with the “ACAACAACAA” motif, which is specific for adult enhancer factor 1 and was found to be enriched at Polycomb binding sites in ChIPs for Pc (*62*). These observations enticed us to further examine the relationships between TET and PcG.

First, we compared TET binding to ChIP-seq data in the larval CNS for members of the PRC1 complex, Polyhomeotic (Ph) and Posterior sex combs (Psc), or the PRC2 complex member Enhancer of Zeste [E(z)] (*44*), as well as for the PcG recruiters Pho (*44*), Cg (*63*), and GAF (our ChIP-seq). In line with the above results, we observed that TET colocalizes with Ph, E(z), and Pho on well-characterized Polycomb response elements (PREs), such as on the *engrailed* (*en*) locus (Fig. 3B). Plotting the ChIP-seq signals for each of these factors across a ±3-kb region surrounding TET peaks, we found that Ph, Psc, E(z), Pho, GA,F and Cg colocalized with TET, with stronger TET peaks associated with stronger binding for these factors (Fig. 3C), although this correlation appeared weaker with E(z). On the other hand, analyses of public ChIP-seq datasets of the architectural proteins Centrosomal protein 190kD (CP190) and CCCTC-binding factor (CTCF) (*64*), which mark boundary/insulator elements, indicate no association of TET with these proteins, in particular for the top 20% TET peaks. Still, when we analyzed the strength of the peaks for PcG proteins or recruiters, we found that those overlapping with TET showed similar strengths as compared to the whole set of peaks, with no marked bias toward weak or strong peaks (fig. S5A). Although considering a direct overlap between model-based analysis of ChIP-seq (MACS2)–defined narrow peaks for TET and PcG proteins, we found that 53% of TET peaks colocalized with Psc peaks and 49% with Ph but only 12% with E(z) (Fig. 3D). These results suggest that there is a stronger overlap between TET and PRC1 (Ph or Psc) than with PRC2 [E(z)].

When we examined the colocalization of TET with PRC1, PRC2, and other chromatin features defined by K-mean clustering, we found distinct patterns of bindings (Fig. 3E). Notably, around 9% of TET peaks colocalized with PRC1 and PRC2 at broad H3K27me3 and H2AK118ub-enriched regions (cluster 2), which correspond to typical large PcG-repressed domains (*42*, *43*), suggesting that TET binds to PREs. Besides, around 18% of TET peaks coincided with PRC1 but not with E(z) on regions enriched in H2AK118ub (cluster 3). This cluster was also enriched in active histone modification marks such as H3K27ac or H3K4me3 and H3K4me1 (*45*, *48*) and likely reflects developmental enhancers, which were previously shown to bind PRC1 and PcG recruiters in larval eye disc and S2 cells (*47*, *65*). Along that line, using a combination of chromatin features to distinguish housekeeping enhancers (H3K4me3 high, H3K4me1 low, and enriched CP190) versus developmental enhancers (H3K4me1 high, H3K4me3 low, and depleted for CP190) (*66*), we found that, like Ph and unlike E(z), TET is specifically enriched at developmental enhancers (fig. S5B).

As the larval CNS is composed of many cell types, it is possible that the above analyses do not reflect the concomitant binding of TET and PcG proteins. Therefore, to assess whether TET colocalizes with PcG on chromatin in vivo, we analyzed the pattern of binding of TET, Ph, and E(z) on polytene chromosomes by immunostaining. As TET is not expressed in the larval salivary gland (fig. S6), we ectopically expressed a hemagglutinin (HA)–tagged version of TET in these cells. In line with our ChIP-seq results, we observed discrete TET signals on all chromosome arms, confirming that *Drosophila* TET binds chromatin (fig. S6). Furthermore, we found that some TET-positive bands colocalized with Ph as well as with E(z) (Fig. 3, F to I and K). TET was more frequently associated with Ph than E(z). Reminiscent of the above results, quantifications on polytene chromosome immunostainings showed that 46% of Ph bands colocalized with TET against 18% for E(z) (Fig. 3L). In sum, these data show that TET is present at PREs of Polycomb-repressed domains and at regulatory regions of active genes bound by PRC1 but not by PRC2.

### PRC1 but not PRC2 is required for the catalytic-independent activation of gene expression by TET

**Fig. 4.**
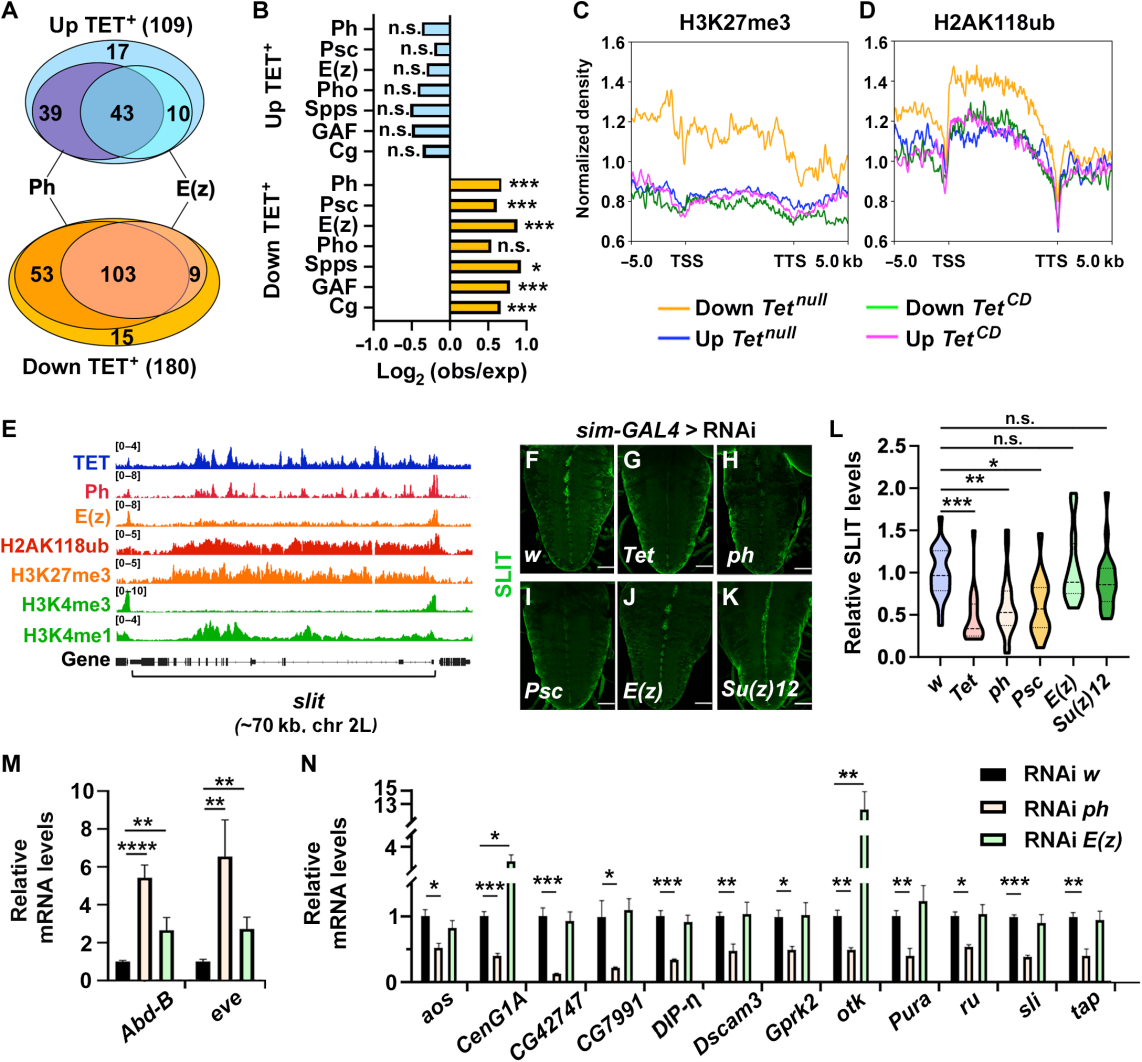
PRC1 but not PRC2 is required for the activation of TET target genes. (**A**) Venn diagrams showing the number of TET^+^ genes up- or down-regulated in *Tet^null^* larval CNS and bound by Ph and/or E(z). (**B**) Peak enrichment of the indicated factors among up- or down-regulated genes bound by TET as compared to random sets of genes. Fisher’s exact test, *P < 0.01; ***P < 0.0001. (**C** and **D**) Profiles of H3K27me3 (C) and H2AK118ub (D) CUT&RUN signals in wild-type larval CNS for TET-bound genes activated or repressed in *Tet^null^* or *Tet^CD^* mutants. (**E**) IGV tracks showing the binding of TET, Ph, and E(z) and the histone modification profiles as indicated, along the *slit* gene. (**F** to **K**) Immunostainings showing Slit expression in the VNC of third instar larvae expressing an RNAi against *white* (*w*) (F), *Tet* (G), *ph* (H), *Psc* (I), *E(z)* (J), or *Su(z)12* (K) in the ventral midline glial cells (*sim-GAL4* driver). Scale bars, 50 μm. (**L**) Corresponding quantifications of Slit levels in the ventral midline. One-way ANOVA test, *P < 0.05; **P < 0.01; ***P < 0.001. (**M** and **N**) RT-qPCRs showing the relative expression of *Abd-B* and *eve* (M), two targets of PcG-mediated repression, and of several target genes activated by TET independently of its enzymatic activity (N), in dissected CNS from *tub-GAL4, tub-GAL80^ts^* third instar larvae expressing an RNAi against *w*, *ph*, or *E(z)* since the early second larval stage. RT-qPCRs were performed in quadruplicates. One-way ANOVA test, **P < 0.01; ***P < 0.001.

To assess the potential functional relationship between TET and PcG proteins, we then focused on TET target genes. We found that 86% (156 of 180) and 62% (112 of 180) of TET-bound genes down-regulated in its absence were bound by Ph or E(z), respectively (Fig. 4A). Similarly, 75% (82 of 109) and 48% (53 of 109) TET-bound genes up-regulated in its absence were bound by Ph or E(z). As compared to random gene sets, PRC1 and PRC2 as well as PcG recruiters were strongly enriched on TET targets down-regulated in the TET absence but not on up-regulated ones (Fig. 4B). Besides, CUT&RUN experiments for H3K27me3 and H2AK118ub in wild-type larval CNS showed that the histone marks deposited by PRC2 and PRC1 were present at higher levels among genes down-regulated in the absence of TET as compared to up-regulated ones (Fig. 4, C and D). H3K27me3 enrichment was observed both upstream of the promoter and within the transcribed regions (with a sharp drop around the TSS and after the TTS), whereas H2AK118ub enrichment was limited to the transcribed region of the gene. These two marks were also higher in *Tet^null^* down-regulated genes as compared to *Tet^CD^* deregulated genes. Moreover, among *Tet^null^* down-regulated genes, those controlled in a catalytic-independent manner displayed higher levels of H3K27me3 and H2AK118ub (fig. S7). Together, these observations indicate that PcG proteins are more specifically associated with genes activated by TET and raise the possibility that they are involved in the catalytic-independent activation of gene expression by this factor. Actually, upon closer examination of PRC1 and PRC2 peaks on the genes activated by TET, we found that TET peaks colocalized with a Psc or a Ph peak in more than 75% of the cases but only in ~25% of the cases with E(z). Conversely, more than 52% of Psc or 55% of Ph peaks and less than 22% of E(z) peaks overlapped with TET (fig. S8). This suggests again a tighter association between TET and PRC1 than with PRC2 and possibly distinct functions for these two complexes in the regulation of TET target gene activation.

To test these hypotheses, we first asked whether PRC1 and/or PRC2 regulate *slit* expression, which does not require TET enzymatic activity (Fig. 1, A to D, and tables S1 and S2). We observed the binding of TET, Ph, and E(z) on this gene (Fig. 4E). Moreover, the *slit* locus was decorated with PcG-associated marks (H3K27me3 and H2AK118ub) as well as with activating marks (H3K4me1 and H3K4me3). In line with a cell-autonomous regulation of *slit* expression by TET in the ventral midline glial cells, RNAi-mediated knockdown of TET in these cells using the *single-minded-GAL4* (*sim-GAL4*) driver led to a decrease in Slit levels (Fig. 4, F, G, and J). Furthermore, the reexpression of TET in these cells in a *Tet^null^* background was sufficient to restore Slit expression (fig. S9). Notably, the knockdown of PRC1 components *ph* or *Psc* in the ventral midline glial cells also caused a reduction in Slit expression (Fig. 4, H, I, and L), whereas the knockdown of PRC2 subunits *E(z)* or *Su(z)12* had no effect (Fig. 4, J to L). These results suggest that PRC1 acts independently of PRC2 and promotes TET-induced *slit* activation.

To extend our analysis of Ph and E(z) impact on other TET targets in the larval CNS, we made use of the ubiquitous *tubulin-GAL4* (*tub-GAL4*) driver combined with *tub-GAL80^ts^* to bypass the early lethality associated with *ph* or *E(z)* loss and inhibit their expression by RNAi only during the larval stages. Under these conditions, we achieved a strong reduction in Ph or E(z) expression in the larval CNS (fig. S10). In addition, reverse transcription followed by quantitative polymerase chain reaction (RT-qPCR) analyses showed that *Abdominal-B* (*Abd-B*) and *even skipped* (*eve*), two known targets of PcG-mediated repression, were strongly up-regulated in the CNS in response to the knockdown of either *ph* or *E(z)* (Fig. 4M), indicating that their function was efficiently impaired. Yet, in line with the above results, we still observed a decrease in Slit expression in the ventral midline upon *ph* but not *E(z)* knockdown (fig. S10). When we monitored by RT-qPCR the expression of several genes whose expression is activated by TET independently of its enzymatic activity and which are bound by both Ph and E(z) such as *argos* (*aos*), *Centaurin gamma 1A* (*CenG1A*), *CG42747*, *CG7991*, *Dpr-interacting protein* η (*DIP-*η), *Down syndrome cell adhesion molecule 3* (*Dscam3*), *G protein–coupled receptor kinase 2* (*Gprk2*), *off-track* (*otk*), *Puratrophin-1-like* (*Pura*), *roughoid* (*ru*), *sli*, and *target of Poxn* (*tap*), all of them were down-regulated upon *ph* knockdown but none upon *E(z)* knockdown (Fig. 4N). Actually, *CenG1A* and *otk* expression were even increased in the absence of E(z), suggesting that PRC2 represses their transcription. Therefore, PRC1, but not PRC2, is required for the catalytic-independent activation of TET target genes in the larval CNS.

### TET and Ph interact together and cross-control their recruitment to chromatin

**Fig. 5.**
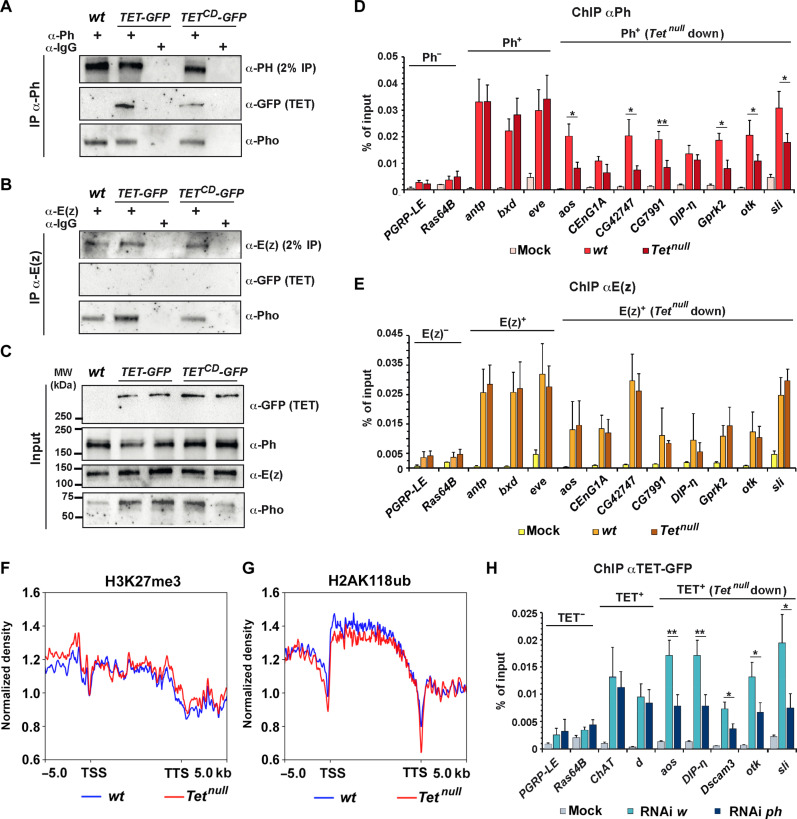
TET and PRC1 interact physically and promote each other recruitment to TET target genes. (**A** and **B**) Coimmunoprecipitation experiments testing the interaction between Ph (A) or E(z) (B) and TET, TET^CD^, or Pho in larval CNS cell extracts from the indicated genotypes. (**C**) Western blots showing the inputs used in (A) and (B). (**D** and **E**) ChIP-qPCRs for Ph (D) or E(z) (E) in dissected CNS from *wt* or *Tet^null^* third instar larval showing their recruitment on canonical PREs (*antp, bxd*, and *eve*) or genes activated by TET independently of its enzymatic activity (*aos*, *CenG1A*, *CG42747*, *C7991 DIP-n*, *Gprk2*, *otk*, and *sli*). *PGRP-LE* and *Ras64B* served as controls without Ph or E(z) binding sites. Mock ChIPs were performed with immunoglobulin G (IgGs). ChIP-qPCRs were performed in triplicates. Student’s *t* test between *wt* and *Tet^null^* conditions, *P < 0.05; **P < 0.01. (**F** and **G**) CUT&RUN signals of TET-activated target genes between their TSS and TTS (±5 kb) for H3K27me3 (F) and H2AK118ub (G) in wild-type or in *Tet^null^* larval CNS. (**H**) ChIP-qPCRs for TET-GFP in dissected CNS from *tub-GAL4, tub-GAL80^ts^*, and *TET-GFP* third instar larvae expressing an RNAi against *w* or *ph* since the second larval stage. Analysis of TET recruitment to genes containing a TET binding site which are not regulated by TET (*ChAT* and *d*) or activated by TET independently of its enzymatic activity (*aos*, *DIP-n*, *Dscam3*, *otk*, and *sli*). *PGRP-LE* and *Ras64B* served as controls that do not contain TET binding sites. Mock ChIPs were performed with IgGs. ChIP-qPCRs were performed in triplicates. Student’s *t* test was performed between *RNAi w* and RNAi *ph* conditions. *P < 0.05; **P < 0.01. MW, molecular weight.

Considering the above results, we performed coimmunoprecipitation experiments using third instar larval CNS protein extracts to test whether TET interacts physically with Ph and/or E(z) in vivo. As shown in Fig. 5 (A to C), an antibody directed against Ph coprecipitated both TET and the PcG recruiter Pho (*43*). In contrast, an antibody directed against E(z) still coprecipitated Pho but not TET. Of note, Ph but not E(z) also interacted with TET CD, indicating that the interaction does not require TET catalytic activity and that the point mutation does not disrupt TET-PRC1 interaction. Hence, these results suggest that TET specifically interacts with PRC1 but not with PRC2.

We hypothesized that TET might promote PRC1 recruitment and the deposition of H2AK118ub at the genes it activates, while it would not affect PRC2 binding and H3K27me3 levels. To test this possibility, we first performed ChIP-qPCR for Ph and E(z) in wild-type or *Tet^null^* larval CNS to assess their binding to chromatin. Consistent with our hypothesis, Ph binding to TET targets was significantly reduced in six of eight cases in the absence of TET (Fig. 5D, Student’s *t* test). In contrast, E(z) recruitment was not affected in any case (Fig. 5E). Moreover, the binding of Ph [or E(z)] to PREs of genes not regulated by TET [*Antennapedia* (*Antp*), *even skipped* (*eve*), and *bithoraxoid* (*bxd*)] was not affected by TET loss (Fig. 5, D and E). Second, we performed CUT&RUN experiments to compare the profile of H3K27me3 and H2AK118ub on TET target genes in the CNS of wild-type and *Tet^null^* larvae. In agreement with our hypothesis, H3K27me3 levels remained unchanged on TET-activated (Fig. 5F) or repressed target genes (fig. S11). In contrast, H2AK118ub levels were slightly decreased on down-regulated genes (Fig. 5G) (paired *t* test *P* = 0.011), while no significant change was observed for up-regulated genes (fig. S11) (paired *t* test *P* > 0.05). TET thus contributes to Ph recruitment and H2AK118ub deposition on the genes it activates.

Last, we assessed whether Ph regulates TET recruitment to its target genes (Fig. 5H). We found that TET binding to *Choline acetyltransferase* (*ChAT*) or *dachs* (*d*), which do not contain Ph binding sites and are not regulated by TET, was not affected upon *ph* knockdown. However, we observed a strong reduction of TET binding on genes activated by TET and harboring a Ph binding site, such as *slit*. Of note, *Ph* knockdown did not affect TET expression, and similarly, TET loss did not affect Ph levels (fig. S12). These results indicate that TET and Ph could promote/stabilize each other’s binding to activate TET target genes. Together, these data strongly support the conclusion that TET specifically interacts with PRC1 but not with PRC2 in the larval CNS and are consistent with a model whereby TET cooperates with PRC1 to promote the transcription of its target genes independently of its enzymatic activity.

## DISCUSSION

In contrast to vertebrates and many other species, the *Drosophila* genome does not code for any 5mC DNA methyltransferase and is largely devoid of this epigenetic mark, suggesting that TET may rather act via a noncanonical mode of action in this model organism. In this study, we show that *Drosophila* TET largely acts in a catalytic-independent manner to promote larval CNS development and that it specifically interacts with PRC1 to activate gene expression.

Previous work using *Tet null* or hypomorphic alleles as well as RNAi-mediated knockdown showed that TET expression is critical for larval CNS development (*31*, *35*, *36*). However, the functional importance of TET enzymatic activity was not assessed in these studies. Here, we show that abolishing TET catalytic activity does not recapitulate the CNS phenotypes observed in *Tet null* mutant larvae. The large majority of genes deregulated in *Tet^null^* was not affected in *Tet^CD^*. Moreover, gene set enrichment analysis revealed that TET expression, but not its enzymatic activity, is important for the activation of CNS development–associated processes. In particular, several genes implicated in axon guidance were only deregulated in *Tet^null^*, which is consistent with the axonal projection defects observed specifically in this condition. Similarly, the EGFR pathway, which controls the development of ventral midline glial cells (*51*, *52*), was also affected only in *Tet^null^*. Recent single-cell transcriptomic experiments indicate that *Tet* is enriched in larval and adult astrocytes (*67*–*69*), and several astrocyte markers, such as *Gaba transporter* (*Gat*), *heartless* (*htl*), and *stumps* are down-regulated specifically in *Tet^null^*, suggesting that TET could control the development of this glial cell population in a catalytic-independent manner. The fibroblast growth factor receptor Htl and its adaptor Stumps are required for astrocyte development, and the down-regulation of *Gat* or astrocyte ablation leads to crawling and muscular contraction defects (*70*) as observed in *Tet* mutant larvae (*32*). Moreover, astrocytes are required for axon pruning in the brain mushroom bodies during pupariation (*71*), and TET loss is associated with mushroom body abnormalities in the adult (*37*, *41*). Hence, essential processes underlying CNS formation are controlled by TET independently of its enzymatic activity.

Yet, many genes were deregulated in the CNS of *Tet^CD^* larvae. We cannot rule out that the HRD to YRA mutation in the TET CD might cause unexpected alterations, but it does not seem to modify TET recruitment to DNA, consistent with similar analyses on TET1 in mouse embryonic stem cells (*28*). Thus, TET probably exerts some of its functions via a catalytic-dependent mechanism. Although one might have expected that most genes deregulated in *Tet^CD^* were also affected in *Tet^null^*, there was a limited overlap between the two conditions. This suggests that CNS cells in *Tet^CD^* larvae have followed a different developmental trajectory as compared to the *null* condition. Alternatively, it could reflect a neomorphic behavior of the Tet^CD^ protein, leading to spurious gene deregulation in the CNS. Still, further experiments will be necessary to decipher the TET catalytic mode of action in *Drosophila.* In contrast with previous reports (*33*, *37*), our recent results indicate that TET does not act as a 6mA demethylase (*41*). Along that line, the capacity of a distant fungal TET homolog to target 6mA requires specific amino acids, which are not conserved among metazoan TETs (*40*). It is thus doubtful that TET catalytic function in the larval CNS is mediated by 6mA demethylation. One hypothesis is that it may involve m5C on RNA. *Drosophila* and mammalian TETs can oxidize m5C into hm5C and thereby control gene expression at the posttranscriptional level by regulating transcript stability, mRNA translation, or tRNA maturation (*21*–*24*, *31*, *72*, *73*). Besides, it remains possible that TET targets 5mC DNA in *Drosophila*, as very low levels of 5mC (0.001 to 0.03%) have been observed in its genome (*33*, *41*, *74*–*76*). However, the impact, if any, of 5mC on *Drosophila* genome biology is still elusive. Last, TET might target other yet unknown substrates at the DNA, RNA, or potentially protein levels.

Focusing on TET enzymatic-independent mode of action, our study highlights an unexpected link between TET and PRC1. The integration of our RNA-seq and ChIP-seq results indicates that TET binding mostly promotes gene expression. This conclusion agrees with a study in BG3-c2 cells and adult brains showing that TET is important for neurodevelopmental gene activation and H3K4me3 accumulation (*37*). At the molecular level, Yao *et al*. (*37*) proposed that TET interacts with Will die slowly (Wds, WDR5 in mammals), a component of the H3K4 methylation Complex Proteins Associated with Set1(COMPASS), to activate transcription and demethylate 6mA in gene bodies to prevent Polycomb recruitment and gene repression. While Wds might contribute to TET-induced gene activation in the larval CNS, we found that TET can activate gene expression in an enzymatic-independent manner and that the PcG protein Ph is required in this process. Furthermore, our data show that TET promotes Ph recruitment (and vice versa) and H2AK118ub deposition at the genes it activates. Previous work in mammals showed that TET1 interacts with PRC2 to repress transcription (*26*, *28*, *77*, *78*). In the larval CNS, TET less frequently colocalized with the PRC2 component E(z) than with Ph and Psc, two components of PRC1. Moreover, contrary to Ph, E(z) did not coprecipitate TET, and its knockdown did not impair the expression of TET targets or its recruitment to chromatin. Conversely, TET loss did not seem to modify E(z) binding nor to affect H3K27me3 deposition. It thus appears that TET does not functionally interact with PRC2 in this context. The presence of high levels of both H3K27me3 and H2AK118ub at TET-activated targets could reflect CNS cell type heterogeneity, whereby TET and Ph/PRC1 promote gene expression in some cells, whereas E(z)/PRC2 might repress the same genes in other cells. As *E(z)* knockdown was not sufficient to induce an overexpression of TET targets we tested (except for *CenG1A* and *otk*), other transcriptional regulators are probably required to activate these genes.

Since ~50% of TET peaks colocalized with PRC1 and *ph* knockdown reduced TET binding, TET could be recruited to chromatin together with PRC1 by the combinatorial action of PcG recruiters such as GAF or Cg, which are strong PRC1 interactors (*79*). However, other determinants could be involved. For instance, interactions with ncRNAs might be important for TET/PRC1 recruitment. In mammals, both TET proteins and PRC1 interact with ncRNAs and are recruited to R-loops (*80*–*83*). Furthermore, other transcription factors likely contribute to TET recruitment. Notably, binding sites for Tango are present in ~6% of TET peaks, including in *slit*. As Tango directly activates the expression of *slit* in the ventral midline glia (*84*), it could help target TET/PRC1 to this gene.

Our data bring further evidence that PRC1 can function independently of PRC2 and contribute to gene activation (*46*). Besides the typical implication of both complexes in gene repression (*42*), PRC1 was shown to bind active genes and promote their transcription independently of PRC2 (*45*, *47*–*49*, *85*, *86*)*.* Yet, how PRC1 mediates gene activation is still unclear. It was shown that PRC1 can assist transcription by modulating RNA polymerase II phosphorylation or the pausing-elongation factor Spt5 occupancy (*48*) and can also contribute to specific chromatin loops favoring promoter/enhancer interactions (*47*). It is tempting to speculate that similar mechanisms underlie the Ph-dependent activation of TET target genes. However, further investigations along those lines are hindered by larval CNS cellular heterogeneity and the accumulation of defects over time. It is anticipated that the development of lineage-restricted chromatin profiling approaches and acute depletion techniques will help gain a finer resolution of TET and PRC1 mode of action in gene activation in vivo.

In conclusion, our work brings strong evidence that TET acts in an enzymatic-independent process to control *Drosophila* CNS development and reveals an unexpected link between TET and PRC1 for gene activation in vivo. Given the conservation of these factors across evolution and their multiple roles during normal development and in diseases, it will be interesting to decipher more precisely the molecular mechanisms underlying their mode(s) of cooperation. Our study also underlines the necessity to consider the noncatalytic functions of epigenetic enzymes to fully embrace their mode of action in normal and pathological situations.

## MATERIALS AND METHODS

### Fly strains and husbandry

The following *D. melanogaster* strains were used: *w^1118^*, *Tet^null^* (*31*) (maintained over *TM6B,Tb*), *Tet-GFP, Tet^CD^-GFP* (*Tet^CD^)* (*41*), *UAS-RNAi white* (Bloomington, BL33623), *UAS-RNAi ph* (Vienna *Drosophila* Resource Center, VDRC50028), *UAS-RNAi E(z)* (BL33659), *UAS-RNAi Psc* (BL31611), UAS-RNAi *Su(z)12* (BL31191), *UAS-RNAi Tet* (BL62280), *sim-GAL4* (BL9150), *sgs3-GAL4* (BL6870), *tub-GAL4*, and *tub-GAL80^ts^* (BL86328). *UAS-HA-Tet* transgenic flies were generated by cloning the full-length *Tet* cDNA (NP_001261344.1) in frame with an N-terminal 3×HA tag into the pUASTattB vector. The resulting plasmid was used by the Centro de Biologia Molecular (CBM) *Drosophila* Transgenesis Facility (Madrid) to generate transgenic flies using the phiC31-based integration system (*87*). Unless otherwise specified, stock maintenance and sample collection were performed using classic fly medium [organic corn flour (75 g/liter), dry yeast (28 g/liter), sucrose (40 g/liter), agar (8 g/liter), and Moldex 20% (10 ml/l)] with a 12-hour dark:light cycle at 25°C. For *ph* and *E(z)* RNAi knockdown experiments, *UAS-RNAi* flies were crossed to *tub-GAL4* and *tub-GAL80^ts^* flies at 18°C. Following 8 hours of egg laying, adult flies were discarded, and the tubes were kept at 18°C for 96 hours before being transferred at 29°C for 48 hours before larval collection.

### In vitro enzymatic assay

TET enzymatic activity was measured using an enzyme-linked immunosorbent assay–based plate assay for DNA hydroxymethylation essentially as described in (*88*). Briefly, the wild type or mutated version of *Drosophila* TET CD was cloned in pET28a expression vector, and the corresponding His-tagged protein was overexpressed in *Escherichia coli* BL21 (DE3) CodonPlus RIL cells and purified on nickel–nitrilotriacetic acid agarose beads as indicated in (*41*). Recombinant TET protein (2 μM) was incubated with 400 ng of biotin-labeled double-stranded DNA substrate [prepared by PCR with 5-methyl-dCTP (2'-deoxy-5-methylcytidine 5'-triphosphate) (5mdCTP) instead of dCTP to methylate all its cytosines] in reaction buffer [50 mM Hepes (pH 6.8), 100 μM ammonium ion(II) sulfate hexahydrate, 1 mM α-ketoglutarate, 1 mM ascorbic acid, and 50 mM NaCl] at 37°C. Reaction was stopped at different time points by adding NaOH, and biotinylated DNA substrate was incubated in an avidin-coated 96-well plate (Sigma-Aldrich) for 1.5 hours. Rabbit anti-5hmC antibody (1:10,000, Active Motif) and goat anti-rabbit horse radish peroxidase (HRP)–conjugated secondary antibody (1:5000, GE Healthcare) were used to measure 5hmC levels by enhanced chemiluminescence (ECL) (Thermo Fisher Scientific) on a CLARIOstarPlus (BMG Labtech).

### Immunostainings and imaging

For CNS immunostaining, wandering third instar larvae were dissected in 1× phosphate-buffered saline (PBS) and fixed for 30 min in PBS containing 4% paraformaldehyde (PFA). Fixed samples were washed twice with PBS and for 30 min with PBS–0.3% Triton X-100 (PBT) before being preincubated for 1 hour in PBT–1% bovine serum albumin (BSA; Sigma-Aldrich). Samples were incubated overnight at 4°C with primary antibody diluted in PBT–1% BSA, washed three times for 15 min in PBT, and incubated with respective secondary antibodies diluted in PBT–1% BSA for 2 hours at room temperature or overnight at 4°C. Samples were washed in PBT, and larval CNS were further dissected and mounted in VECTASHIELD–4′,6-diamidino-2-phenylindole (DAPI) (Vector Laboratories).

For polytene chromosome preparation, *UAS-HA-Tet* flies were crossed to *salivary gland secretion 3-GAL4* (*sgs3-GAL4*) flies at 18°C. Third instar larvae were collected, and salivary glands were dissected in PBS and fixed for 30 s in PBS, 1% Triton X-100, and 3.7% PFA and for 2 min and 30 s in PBS, 3.7% PFA, and 50% acetic acid. Salivary glands were squashed between a coverslip and a poly-l-lysine–treated slide to spread chromosomes. Slides were then snap-frozen in liquid nitrogen. Coverslips were quickly removed, and slides were washed twice for 15 min in PBS before incubation for 1 hour in blocking solution (PBS and 1% BSA). Antibodies diluted in blocking solution were then incubated with the slides at 4°C overnight in a humid chamber. Slides were washed twice in washing solution (PBS, 300 mM NaCl, 0.2% NP-40, and 0.2% Tween 20) before being incubated for 2 hours at room temperature with the appropriate secondary antibodies diluted in the blocking solution. Chromosome preparations were then washed twice in the washing solution and mounted in VECTASHIELD-DAPI (Vector Laboratories).

The following antibodies were used: mouse anti-Slit (dilution 1:50; Developmental Study Hybridoma Bank, DSHB C555.6D), mouse anti-Fas3 (1:20; DSHB 7G10), rat anti-HA (1:500; Sigma-Aldrich, #11867423001), goat anti-Ph (1:500) (*89*), rabbit anti-E(z) (1:500) (*90*), donkey anti-rabbit, anti-mouse, or anti-goat Alexa Fluor 488 (1:1000; Invitrogen), and donkey anti-rat or anti-mouse Cy3 (1:1000; Jackson ImmunoResearch). Images were acquired using a Zeiss SP8 confocal microscope.

### RNA-seq and data analysis

Total RNA was isolated from 30 third instar larval brains dissected in ice-cold PBS using TRIzol extraction. RNA quality and concentration were assessed with the Agilent TapeStation and the Qubit device. Libraries were prepared using the Illumina TruSeq RNA Library Prep kit and sequenced by Novogene (Cambridge, UK) on Novaseq 6000 (paired-end, 150 bp). Reads were filtered and trimmed to remove adapter-derived or low-quality bases using Cutadapt and checked again with FASTQC. Illumina reads were aligned to the *Drosophila* reference genome (dm6 Ensembl release 70) with Hisat2. Read counts were generated for each annotated gene using HTSeq-Count. RPKM (reads per kilobase of exon per megabase of library size) values were calculated using Cufflinks. Read normalization, variance estimation, and pairwise differential expression analyses with multiple testing corrections were conducted using the R Bioconductor DESeq2 package. Gene set enrichment analyses were performed with Pangea (*91*), using all the genes expressed in the larval CNS as a background universe. Enrichment in Gene Ontology (GO) terms and pathways [Kyoto Encyclopedia of Genes and Genomes (KEGG), Panther, Reactome] was considered significant for a *P* value < 0.01 using the Benjamini-Hochberg correction.

Violin plots, histograms, volcano plots, and GO enrichment dot plots were generated using the R package “ggplot2.” Heatmaps were obtained using the R package “plotheatmap.” Statistical tests were realized with R or GraphPad Prism.

### ChIP-seq and ChIP-qPCR

ChIP-seq and ChIP-qPCR were performed as previously described (*92*) with minor modifications. Briefly, for ChIP-seq, ~400 third instar larvae brains were dissected per replicate in ice-cold PBS, and experiments were performed in duplicates for each genotype. For ChIP-qPCR, ~100 CNS were used per replicate, and experiments were performed in triplicates. Samples were fixed for 15 min in 1.8% PFA with shaking before quenching for 5 min by adding glycine to a final concentration of 350 mM. Tissues were lysed in 1% SDS lysis buffer (140 mM NaCl, 15 mM Hepes, 1 mM EDTA, 0.5 mM EGTA, 1% Triton X-100, 0.5 mM dithiothreitol, 0.1% sodium deoxycholate, 10 mM sodium butyrate, and cOmplete EDTA-free protease inhibitor cocktail (Merck)] before sonication using a Bioruptor pico device (Diagenode) with the following settings: 18 cycles, 30 s on/30 s off. The size of DNA fragments was confirmed on agarose gel (200 to 300 bp). Samples were precleared with a 50/50 mix of protein A agarose beads (Sigma-Aldrich, P7786) and protein G Sepharose Fast Flow beads (Sigma-Aldrich, P3296) for 4 hours at 4°C. Beads were removed, and anti-GFP (Abcam Ab290), anti-Ph (*89*), or anti-E(z) (*90*) antibodies diluted in 1:200 were added and incubated overnight with the samples. Beads were added for another incubation of 4 hours at 4°C and then washed. DNA was de-crosslinked at 65°C before purification with phenol:chloroform (Sigma-Aldrich, 77617) and precipitation overnight in ethanol. For ChIPs followed by qPCR, inputs were diluted in 1:100 and samples in 1:10. qPCR reactions were performed with the SsoFast EvaGreen reagent (Bio-Rad) on a LightCycler 480 Instrument II (Roche Life Science). Primer sequences used for ChIP-qPCR are provided in table S4. For ChIP-seq, libraries were prepared using the NEBNext Ultra II DNA Library Prep Kit for Illumina (New England Biolabs, E7103) following the manufacturer’s instructions. Last, sequencing was performed by Novogene (Cambridge, UK) on a Novaseq 6000 device (paired-end, 150 bp).

### Cleavage under targets and release using nuclease

CUT&RUN was performed as previously described (*93*). For each sample, around 30 third instar larval CNS were dissected in cold PBS. All experiments were performed in duplicates. Tissues were bound to BioMag Plus Concanavalin A–conjugated magnetic beads (Polysciences Inc.) before blocking and permeabilization in dbe + buffer containing 20 mM Hepes, 150 mM NaCl, 0.1% BSA, 0.5 mM spermidine, 2 mM EDTA, 5% digitonin, and cOmplete EDTA-free protease inhibitor cocktail. The antibodies (table S4) were diluted (1:100) in the same buffer before overnight incubation at 4°C. Samples were washed with dbe + buffer and then incubated for 1 hour at room temperature with Proteins A and G fused to Micrococcal Nuclease produced in *E. coli *for CUT&RUN Assays (CUTANA pAG MNase) (EpiCypher, #15-1016) diluted in dbe + buffer. After a wash in dbe + buffer, DNA was cleaved in wash + C buffer on ice for 30 min. The cleavage reaction was stopped, and samples were treated with ribonuclease A for 30 min at 37°C. After 2 hours of proteinase K treatment at 50°C, DNA was recovered using Ampure XP beads (Beckman Coulter). Library preparation and sequencing were performed as detailed for ChIP-seq.

### Processing of ChIP-seq and CUT&RUN data

Sequencing adapters were removed using TrimGalore (v0.6.6, --paired, --stringency 6). Resulting reads were mapped to the dm6 genome using bowtie2 (v2.4.2) reporting at most one hit for each read (the best one according to Mapping Quality score). Peaks were called using MACS2 (v2.2.7.1) to capture narrow (-q 0.05 -g dm) and broad peaks (-q 0.05 -g dm –broad –broad-cutoff 0.1). As a control, we used a ChIP with immunoglobulin G (IgGs) for each condition except for TET-GFP and TET^CD^-GFP, which were compared to ChIPs with GFP in a *w^1118^* background. Final peak calling was done on merged replicates.

Chromatin state predictions were done using CHROMHMM software (*60*). We used the following public dataset: STARR-seq of BG3-c2 enhancers GSE49809 (*94*); ChIP-seq Cg GSE77582 (*63*); ChIP-seqs CP190 and CTCF GSE146752 (*64*); ChIP-seqs E(z), Ph, Psc GSE102339, and Pho GSE102338 (*44*); ChIP-seq H3K36me3 PRJNA494709 and RNA PolII PRJNA564118 (*58*); ChIP-seq H3K4me1 GSE126985 (*47*); Dam-ID HP1a GSE109495 (*59*). Signal heatmaps and profiles were provided with plotheatmap() and plotprofile() functions from the deeptools software suite, respectively. The Meme suite was used to find enriched motifs in peaks by applying the STREME function.

### Reverse transcription followed by quantitative polymerase chain reaction

For RT-qPCR, RNA samples were prepared from dissected larval CNS using an RNeasy kit (Qiagen) with an additional on-column deoxyribonuclease (DNAse) treatment with the RNase-Free DNase Set (Qiagen). Reverse transcription was performed with SuperScript IV Reverse Transcriptase (Thermo Fisher Scientific) on 100 ng of RNA, using a mix (1:1) of random primers (Promega) and oligo dT (Promega) according to the manufacturer’s instructions. qPCRs were performed with the SsoFast EvaGreen reagent (Bio-Rad) on a LightCycler 480 Instrument II (Roche Life Science). Primer sequences used in RT-qPCR are provided in table S4. qPCR data were analyzed with the ∆∆Ct method, and gene expressions were normalized to *glyceraldehyde-3-phosphate dehydrogenase 1 (gadph1)*. All experiments were performed in biological quadruplicates.

### Coimmunoprecipitations

Immunoprecipitation reactions were carried out as described in (*63*). Proteins from dissected third instar larval CNS were extracted in cold PBS supplemented with cOmplete EDTA-free protease inhibitor cocktail (Merck). Tissues were crushed with a motorized pestle in a buffer containing 25 mM Hepes, 0.1 mM EDTA, 12.5 mM MgCl_2_, 10% glycerol, 0.01% NP-40, 10 mM NaCl, and a cOmplete EDTA-free protease inhibitor cocktail before sonication with the Bioruptor pico device (Diagenode) (settings: 3 cycles, 30 s on/30 s off). Protein concentration was assayed by the DC Protein Assay (Bio-Rad), and 1 mg of proteins was used per reaction. Proteins were precleared with a 50:50 mix of protein A agarose beads (Sigma-Aldrich) and protein G Sepharose Fast Flow beads (Sigma-Aldrich) for 4 hours at 4°C. After bead elimination, samples were incubated with anti-Ph or anti-E(z) (1:200) overnight at 4°C. Beads were then added to the samples for 4 hours at 4°C. After three washes in the same buffer, proteins were boiled directly in 4× Laemmli sample buffer before being subjected to Western blot analysis (see below).

### Western blots

Proteins were loaded on 7.5% or 4 to 20% Mini-PROTEAN TGX Stain-Free Gels (Bio-Rad) after the addition of 4× Laemmli sample buffer and denaturation at 95°C for 5 min. After migration, proteins were transferred to a nitrocellulose blotting membrane (Amersham). Membranes were washed in tris-buffered saline (TBS) and then blocked for 1 hour at room temperature in blocking solution (TBS, 10% milk, and 0.1% Tween 20). The primary antibodies were diluted in washing buffer (TBS, 2% milk, and 0.1% Tween 20) and incubated with the membranes overnight at 4°C at the following concentrations: anti-Ph (1:1000), anti-E(z) (1:1000), anti-Pho [1:1000, a gift from J. Kassis; (*44*)], anti-GFP (1:1000: Takara, JL-8), and anti-tubulin (1:1000; Sigma-Aldrich, DM1A) (table S4). Membranes were washed three times in washing solution and incubated with the appropriate HRP-conjugated secondary antibodies for 2 hours at room temperature. Detection was performed using the Clarity Western ECL substrate (Bio-Rad) according to the manufacturer’s instructions, and the signals were analyzed with the ChemiDoc MP Imaging System (Bio-Rad).
