# Supplementary material for: Drosophila TET acts with PRC1 to activate gene expression independently of its catalytic activity
Source: Sci Adv. 2024 May 3;10(18):eadn5861. doi: 10.1126/sciadv.adn5861 (PMC11068012; doi:10.1126/sciadv.adn5861)
Supplement: Supplementary file 1 — Figs. S1 to S12 Legends for tables S1 to S4 [file sciadv.adn5861_sm.pdf]

Science Advances
AAAS

Supplementary Materials for

Drosophila TET acts with PRC1 to activate gene expression independently of its catalytic activity

Guerric Gilbert *et al.*

Corresponding author: Lucas Waltzer, lucas.waltzer@uca.fr; Laurence Vandel, laurence.vandel@uca.fr

Sci. Adv. **10**, eadn5861 (2024)
DOI: 10.1126/sciadv.adn5861

The PDF file includes:

Figs. S1 to S12
Legends for tables S1 to S4

Other Supplementary Material for this manuscript includes the following:

Tables S1 to S4

Figure S1

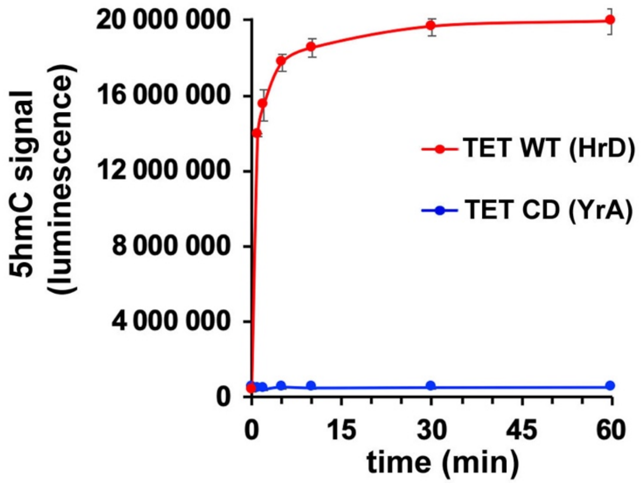

Fig. S1. Mutation of TET iron binding motif abolishes its enzymatic activity *in vitro*. The levels of 5hmC produced at different time points following the incubation of 5mC DNA with recombinant TET catalytic domain carrying a wild type (WT, red) or mutated (CD, blue) iron binding motif are represented. Error bars denote standard deviations from 3 independent experiments.

Figure S2

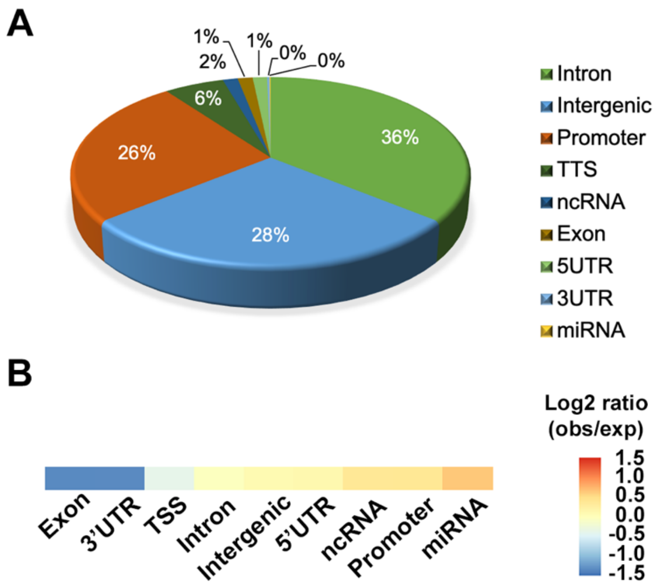

Fig. S2. Analyses of TET^{CD}-GFP ChIP-seq peaks repartition in the *Drosophila* genome. (A) Pie chart of TET^{CD}-GFP peaks distribution according to RefSeq genomic annotations. (B) Heatmap showing TET^{CD}-GFP enrichment according to genomic features.

Figure S3

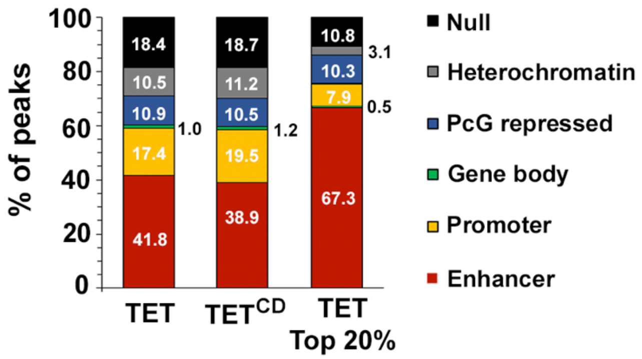

Fig. S3. TET essentially binds to enhancer regions. The proportions of TET and TET^{CD} ChIP-seq peaks associated with each of the 6 chromatin states as defined using ChromHMM in Fig. 2E are represented.

Figure S4

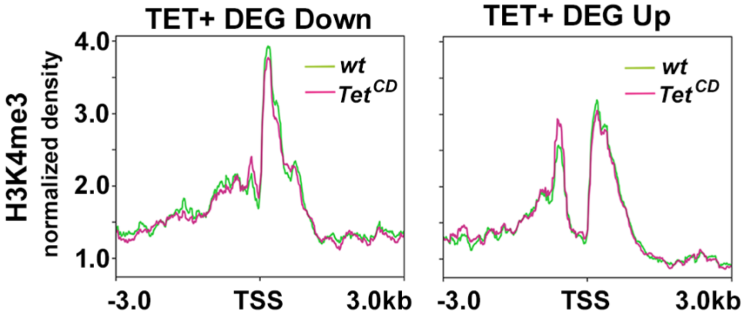

Fig. S4. The loss of TET enzymatic activity does not alter the H3K4me3 profile of its target genes. H3K4me3 CUT&RUN profiles in wild type (green) or *Tet*^{CD} (pink) condition of genes bound by TET and down-regulated (left panel) or up-regulated (right panel) in the CNS *of Tet*^{CD} larvae. TSS: Transcription Start Site.

Figure S5

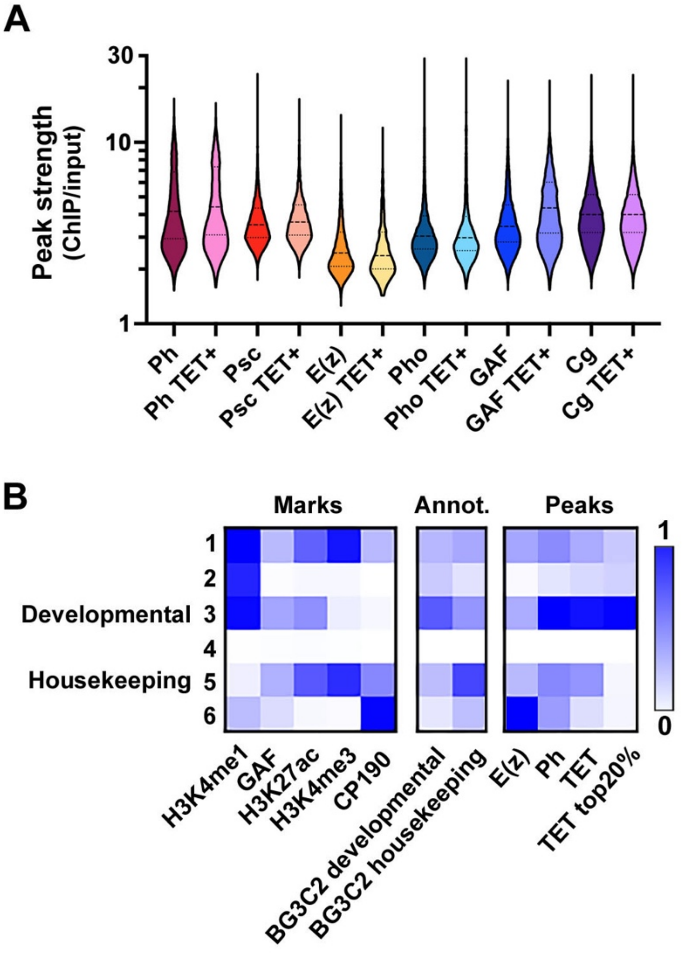

Fig. S5. Polycomb protein peak strength is not dependent on TET binding and TET is associated with developmental enhancers. (A) Violin plot of peak strength showing total peaks or peaks that colocalize with TET (TET+) of the indicated PcG proteins (Ph, Psc, E(z)) or recruiter proteins (Pho, GAF, Cg). (B) Heatmap of the CNS larval chromatin states: developmental and housekeeping enhancers were identified with the ChromHMM package using ChIP-seq or CUT&RUN signals for H3K4me1, H3K4me3, H3K27ac, GAF and CP190. States 3 and 5 correspond, respectively, to developmental or housekeeping enhancers, as confirmed by the projection of the dataset of STARR-seq signals (Annot.) from the neuronal cell line BG3-c2 cells (*94*) for these two categories of enhancers.

Figure S6

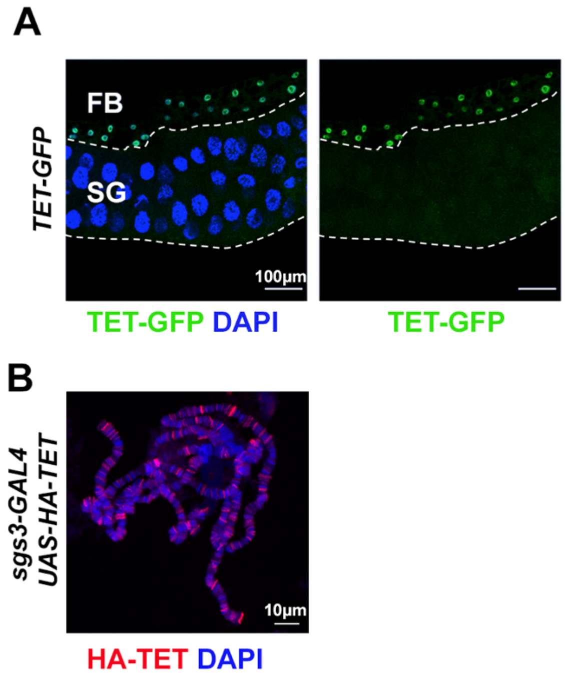

Fig. S6. TET is not expressed in the larval salivary gland and binds chromatin upon ectopic expression. (A) Immunostaining against GFP showing the expression of the endogenous TET protein tagged with GFP (*TET-GFP* knock-in) in the larval salivary gland (SG) and surrounding fat body (FB). Left panel: DAPI and GFP. Right panel: GFP only. (B) Immunostaining against HA on salivary gland polytene chromosomes showing the ectopically expressed HA-tagged TET protein (*sgs3-GAL4; UAS-HA-TET*). DNA was stained with DAPI.

Figure S7

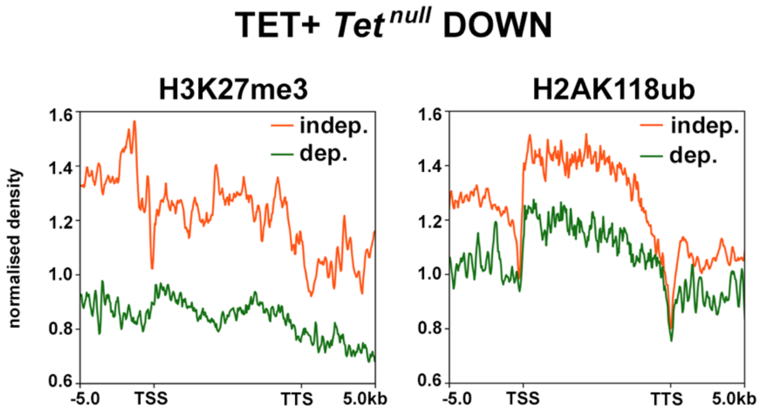

Fig. S7. Genes activated by TET in a catalytic-independent manner display higher levels of H3K27me3 and H2AK118ub. H3K27me3 (left panel) and H2AK118ub (right panel) CUT&RUN signal profiling in the CNS from wild type larvae on genes bound by TET and down-regulated specifically in Tet^{null} (catalytic-independent, orange) or both in Tet^{null} and Tet^{CD} (catalytic-dependent, green). TSS: transcription start site. TTS: transcription termination site.

Figure S8

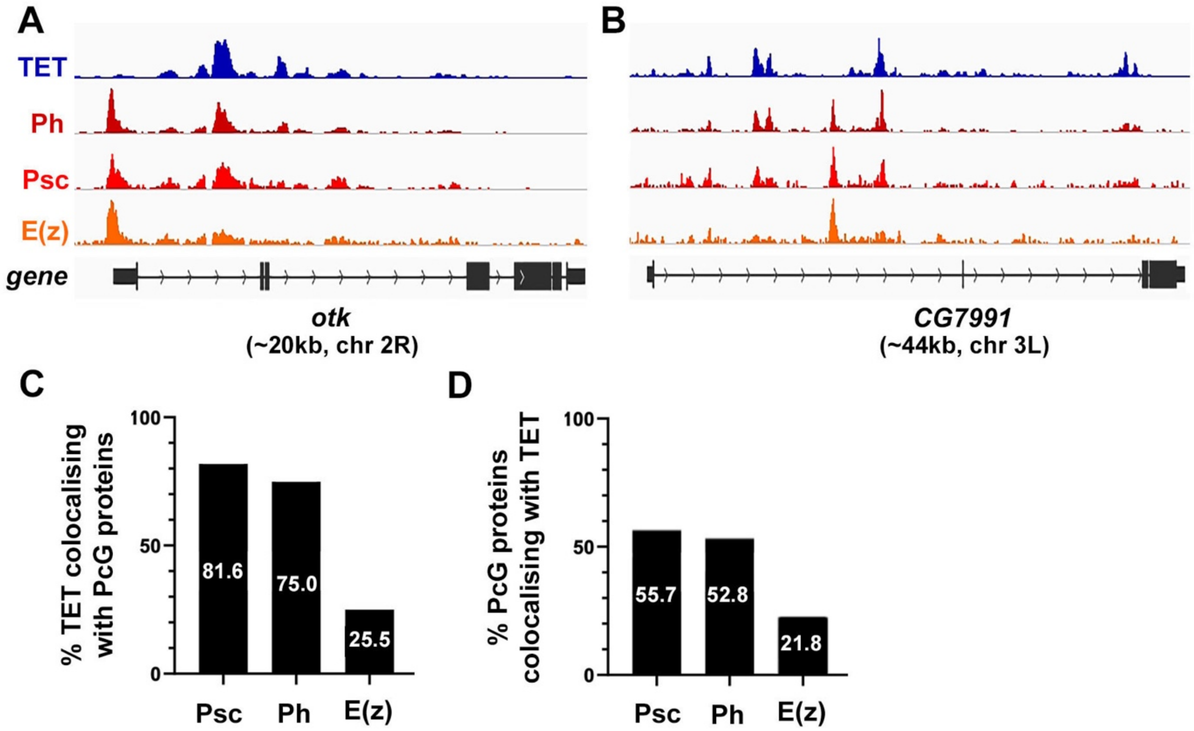

Fig. S8. TET colocalizes preferentially with PRC1 rather than with PRC2. (A, B) IGV tracks showing the binding pattern of TET, Ph and E(z) on *otk* (A) and *CG7991* (B). (C) Proportions of TET peaks aligned with at least one Ph or E(z) peak among TET+ genes down-regulated in *Tet*^{null} larval CNS. (D) Proportions of Psc, Ph or E(z) peaks aligned with at least one TET peak among TET+ genes down-regulated in *Tet*^{null} larval CNS.

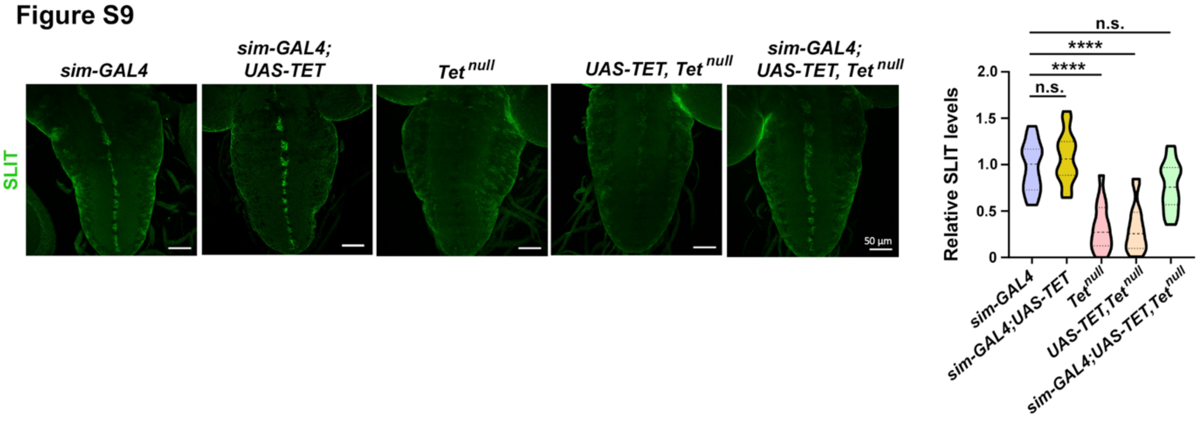

Fig. S9. The re-expression of TET in the ventral midline of *Tet*^{null} larvae is sufficient to restore Slit expression. Left panels: immunostaining showing Slit expression in the ventral nerve cord of third instar larvae of the indicated genotypes. Scale bar, 50 µm. Right panel: quantification of Slit levels in the ventral midline (n=18 per genotype). ****: One-way ANOVA $p<0.0001$.

Figure S10

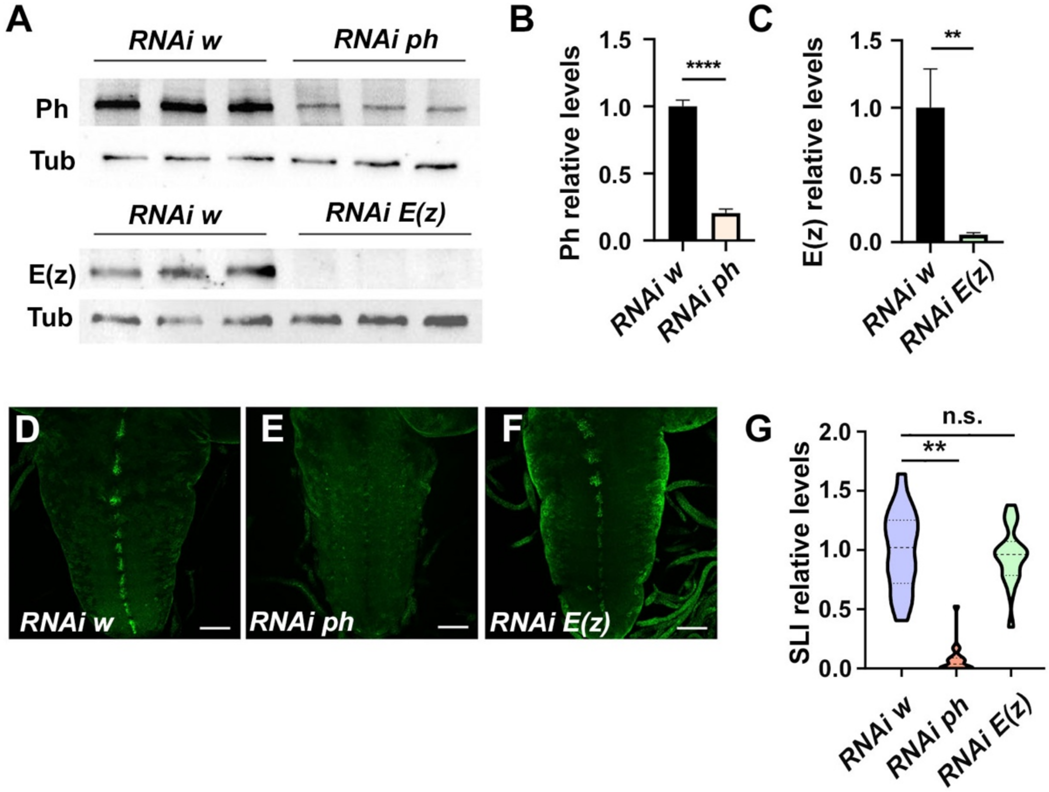

Fig. S10. The knock-down of Ph but not E(z) expression causes a reduction in Slit expression. *tub-GAL4, tub-GAL80*^{ts} individuals carrying *UAS-RNAi* transgene directed against *white* (*w*), *ph* or *E(z)* were raised at 18°C for 104h then switched at 29°C for 48h and their CNS were dissected. (A) Western blots showing the expression of Ph, Tubulin (Tub) or E(z) in the CNS of third instar larvae expressing an RNAi against *w*, *ph* or *E(z)*. (B, C) Corresponding quantifications of Ph (B) and E(z) (C) protein levels relative to Tub expression. Student's t-test ****: p<0.0001, **: p<0.01. (D-F) Immunostaining showing Slit expression in the ventral nerve cord of third instar larvae expressing an RNAi against *w* (D), *ph* (E) or *E(z)* (F). (G) Corresponding quantifications of Slit levels in the ventral midline (n=18 per genotype). Scale bar, 50 µm. One-way ANOVA **: p<0.01, n.s. non-significant.

Figure S11

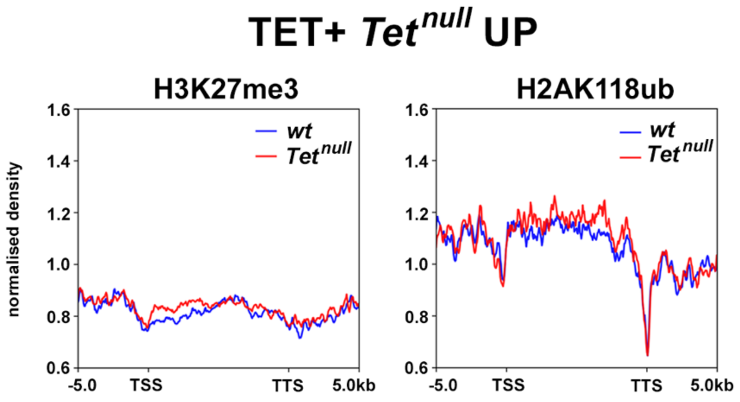

Fig. S11. The H3K27me3 and H2AK118ub profiles of genes bound and repressed by TET are not affected by the loss of TET. H3K27me3 (left panel) and H2AK118ub (right panel) CUT&RUN signal profiling in the CNS from wild type (*wt*, blue) or *Tet*^{null} (red) larvae on genes bound and repressed by TET. TSS: Transcription Start Site. TTS: Transcription Termination Site.

Figure S12

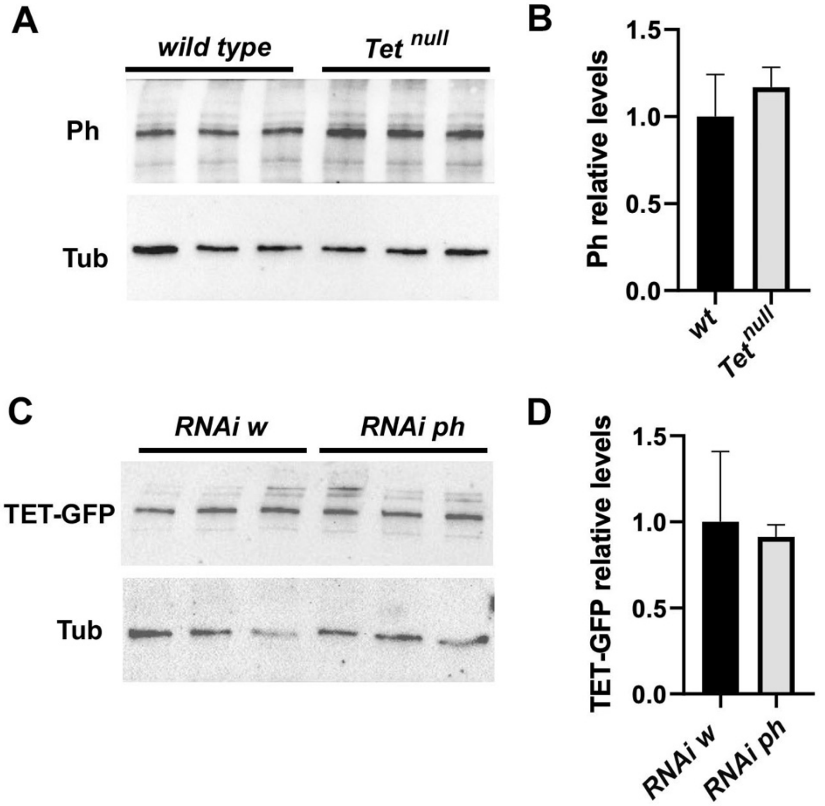

Fig. S12. TET and Ph do not cross-regulate their expression. (A, C) Western blots showing the expression of Ph and Tubulin (Tub) in the CNS of wild type or *Tet*^{null} third instar larvae (A), or the expression of TET-GFP and Tub in the CNS of third instar larvae expressing an RNAi against *w* or *ph*. (B, D) Corresponding quantifications of Ph (B) and TET-GFP (D) protein levels relative to Tub expression.

Table S1. List of differentially expressed genes between *Tet*^{null} and wild type third instar larval CNS.

Table S2. List of differentially expressed genes between *Tet*^{CD} and wild type third instar larval CNS.

Table S3. List of enriched terms for all the genes either deregulated in *Tet*^{null} or *Tet*^{CD} or only deregulated in one or the other case (*Tet*^{null} spe; *Tet*^{CD} spe).

Table S4. List of primers and antibodies used in this study.
